# Deep Shape Features for Predicting Future Intracranial Aneurysm Growth

**DOI:** 10.3389/fphys.2021.644349

**Published:** 2021-07-01

**Authors:** Žiga Bizjak, Franjo Pernuš, Žiga Špiclin

**Affiliations:** Laboratory of Imaging Technologies, Faculty of Electrical Engineering, University of Ljubljana, Ljubljana, Slovenia

**Keywords:** intracranial aneurysm, growth prediction, vascular disease, deep learning, classification, morphologic features

## Abstract

**Introduction:** Intracranial aneurysms (IAs) are a common vascular pathology and are associated with a risk of rupture, which is often fatal. Aneurysm growth is considered a surrogate of rupture risk; therefore, the study aimed to develop and evaluate prediction models of future artificial intelligence (AI) growth based on baseline aneurysm morphology as a computer-aided treatment decision support.

**Materials and methods:** Follow-up CT angiography (CTA) and magnetic resonance angiography (MRA) angiograms of 39 patients with 44 IAs were classified by an expert as growing and stable (25/19). From the angiograms vascular surface meshes were extracted and the aneurysm shape was characterized by established morphologic features and novel deep shape features. The features corresponding to the baseline aneurysms were used to predict future aneurysm growth using univariate thresholding, multivariate random forest and multi-layer perceptron (MLP) learning, and deep shape learning based on the PointNet++ model.

**Results:** The proposed deep shape feature learning method achieved an accuracy of 0.82 (sensitivity = 0.96, specificity = 0.63), while the multivariate learning and univariate thresholding methods were inferior with an accuracy of up to 0.68 and 0.63, respectively.

**Conclusion:** High-performing classification of future growing IAs renders the proposed deep shape features learning approach as the key enabling tool to manage rupture risk in the “no treatment” paradigm of patient follow-up imaging.

## 1. Introduction

Intracranial aneurysms (IAs) are a common cerebrovascular pathology characterized as abnormal bulges forming on the intracranial vessel wall. Most often, they are located near the circle of Willis (approximately 85%) (Schievink, 1997) but also near vessel bifurcations. Despite being highly prevalent (~2–8% of the general population) (Vlak et al., 2011), most IAs do not rupture throughout the lifetime of the patient. In case of rupture, the bleeding from IA may cause hemorrhagic stroke, a serious life-threatening condition with a high fatality rate (50%), while about 66% of those who survive suffer from a permanent neurological deficit.

Increasingly more IAs are accidentally discovered before rupture, during screening for other intracranial vascular diseases. In such situations, the treatment path needs to be determined. Surgical treatments, such as coiling and clipping, are associated with a certain risk of complications. For instance, the results of the ISUIA trial showed that morbidity and mortality were 12.6 and 10.1%, respectively, for surgical clipping and endovascular therapy (Hodes et al., 1959). Furthermore, the risk of complications progressively increases with age (Sedat et al., 2002; Brinjikji et al., 2013).

Due to the rather high risks of surgical treatments and a high volume of cases, the “no treatment” approach with follow-up imaging and IA monitoring seems a possible alternative. Specifically, the imaging is justified when the risk of surgical treatment is higher than the risk of spontaneous rupture (Brinjikji et al., 2016). To date, clinical decisions regarding unruptured IAs and the interval between two imaging sessions are largely based on the intuition of the clinician. The general distrust toward previously published IA growth predictors is based on contradictory research results and low predictive values. For instance, Brinjikji et al. (2016) performed a review and meta-analysis and estimated, based on rupture incidence analysis, that rupture risk for small IAs is 0.8% (aneurysm size <3.9 mm), 1.2% for medium (4–10 mm), 7.1% for large (10–25 mm), and 43.1% for the giant ones (>25 mm). On the contrary, Chien et al. (2019) showed that aneurysm size is not significant factor of future growth.

There is a prompt demand for a more objective decision-making in the management of patients with aneurysms, which may be achieved with the support of computer-aided prognostic tools. In this study, we developed and evaluated such tools to prioritize treatment or extend the surgery waiting periods of patients with aneurysms, based on baseline imaging data. Our primary aim was to answer the following research question: “Can we predict, and with what level of performance, the future aneurysm growth from an angiographic scan acquired prior to rupture?”

## 2. Background

A few quantitative approaches that aim to objectivize the clinical decision-making between longitudinal follow-up IA monitoring or surgery have already been proposed. One popular approach is the PHASES score (Backes et al., 2015), proposed in a study including 557 subjects. The PHASES score provides absolute risks of IA rupture based on six easily retrievable risk factors [population, hypertension, age, size of aneurysm, earlier subarachnoid hemorrhage (SAH) from another IA, and site of IA]. Two years later, the same authors updated the study and proposed the ELAPSS score (Backes et al., 2017). The main factors in the ELAPSS score are the location of the IA, age of the subject, population, size of the aneurysm, shape of the aneurysm, and earlier SAH. Even though several factors are considered, the aneurysm size can contribute up to 55% of the final score.

Although several other studies already questioned the aneurysm size as the key parameter in rupture risk prediction (Clarke, 2008; Sonobe et al., 2010), the PHASES score still attributes the size as having the highest impact on aneurysm rupture risk. First, this is somewhat biased against small aneurysms, namely, these will have little chance of being classified as high risk. However, the small aneurysms also grow and occasionally rupture. Second, the study of 382 patients (Chien et al., 2019) concluded that aneurysm size is not a significant factor of aneurysm growth prediction, whereas growth is considered a surrogate for rupture risk (Villablanca et al., 2013).

Another drawback of aneurysm size is the current measurement procedure. The IA size is usually measured on 2D cross-sections of the 3D image using manual measurement tools. This process does not take into account the full 3D morphology and the measurement objective is ill-defined; size could be ambiguously understood as maximal diameter, transverse diameter, dome height, etc. The required measurement precision is in the range of spatial image sampling (below 1 mm), which is in the range of manual measurement error. Furthermore, the threshold for growth identification is vaguely defined, i.e., from 0.5 to 2 mm. Growth could also be characterized by irregular IA shape developments, which are prone to subjective judgment. To avoid subjective judgment and related measurement errors, an alternative is to use automatic methods for aneurysm growth prediction.

Recent attempts to understand rupture risk and aneurysm growth have taken new approaches, such as using hemodynamic modeling, morphological analysis, and wall inflammation detection (Can and Du, 2016; Hu et al., 2016; Skodvin et al., 2019).

The study on 382 patients (Chien et al., 2019) showed that smoking and hypothyroidism had a large effect on the growth rate of large aneurysms. Aneurysms in patients with multiple IAs were 2.43 times more likely to grow than those in patients with single IAs. The growth rate of large IAs was significantly faster than the growth rate of small IAs (*p*-value of 0.003). Although small IAs were less likely to grow, there is a need to identify those small aneurysms that tend to grow. For instance, patients harboring such IAs should be monitored more frequently. Although there are many clinical, hemodynamic, and geographic data that may aid the future IA growth prediction, the focus of this study is on predicting IA growth based on aneurysm morphology.

Several studies have established (Villablanca et al., 2013; Brown Jr and Broderick, 2014) that simple *in-vivo* measurements of morphologic features such as aneurysm size (HMAX), aspect ratio (AR), non-sphericity index (NSI), size ratio, volume (V), surface area (SA), and others are important factors for assessing rupture risk; however, they are not decisive factors. Liu et al. (2018) have studied the prediction performance of several morphological features, among which vessel size, aneurysm size, perpendicular height, aspect ratio, size ratio, aneurysm angle, aneurysm lobulations, and hypertension achieved a predictive *p*-value of 0.001 or less. They found single cross-sectional feature insufficiently informative for future IA growth prediction. On the other hand, in a study by Chien et al. (2018), only the NSI proved informative for growth prediction [area under receiver operating characteristic (ROC) curve of 0.72], while other features such as HMAX, V, AR, and SA achieved AUC values close to 0.5 (random guess). It seems that univariate models based on crude features of IA morphology may have limited prediction performance.

Neyazi et al. (2020) recently concluded that a high number of established morphological and hemodynamical parameters seem to have little or no effect on the prediction of aneurysm rupture. In their study, they analyzed 21 morphological and 28 hemodynamic parameters. Some of the morphologic parameters that they found significant were the maximal height, volume, area, aspect ratio, convex hull volume, non-sphericity, and gamma of the aneurysm. However, the results showed mediocre performance. Then they constructed a regression model using forward selection and achieved an AUC of 0.75 for rupture prediction. Their study shows that multivariate models, incorporating complementary morphologic parameters describing the IA, may improve the prediction over the univariate models and thus are a promising avenue to explore.

The morphologic features discussed thus far are univariate shape descriptors employed only in the domain of IA analysis. In general, the shape descriptor is considered a concise yet informative vector representation that provides a 3D object with an identification signature as a member of some category. Therefore, shape descriptors can provide much more information than any morphologic feature alone. Recent deep learning-based methods adopted for unstructured data such as point clouds (Qi et al., 2017a) enable the automatic extraction of shape descriptors tailored for a specific task and thus will be in the focus of this study.

## 3. Materials and Methods

### 3.1. Data

Our dataset consisted of CT angiography (CTA) and magnetic resonance angiography (MRA) of 39 patients. The inclusion criteria for this study were as follows: (i) each patient harbored at least one unruptured IA, (ii) the unruptured IAs in these patients were untreated, and (iii) follow-up imaging included at least two angiographic scans with >6 months time difference. This study was based on secondary data. Data were de-identified before being used in this study. The results of this study did not impact patient care.

All images were acquired at University Medical Centre Ljubljana using standard imaging protocols as in the clinical routine. For instance, the matrix size of the CTA scanning protocol was 512 ×512, with an in-plane pixel size of 0.19−0.41 mm, a section thickness of 0.6–1 mm, and a field of view of 180 mm. The matrix of MRA images was 512 ×512, in-plane 0.4 ×0.4 mm spacings, and 60–140 transverse slices with 0.5–0.8 mm thickness. The median difference between the two imaging sessions was 2 years.

The 39 patients had a total of 44 aneurysms. The size of IAs at baseline scan varied from 1.4 mm to 12.2 mm with a median value of 5.01 mm. Hence, the majority of IAs were small to medium size. Follow-up imaging aimed to monitor the morphological changes of the IAs observed at baseline. Namely, each particular IA was labeled either as *growing* or *stable* based on visual comparison of the baseline and follow-up scans by a neurosurgeon with more than 10 years of experience in IA assessment and treatment. Among the 44 IAs, there were 25 labeled as growing and 19 as stable (57 vs. 43%, respectively). Information about the datasets is reported in Table 1.

**Table 1:** Dataset information.

Number of patients (male/female)		**39 (15/24)**
Patient age span (median)		43–85 (67)
Number of intracranial aneurysms		44
Imaging modality (CTA/MRA)		20/24
Aneurysm location	Posterior communicating artery Superior hypophyseal artery Ophthalmic artery	25 6 13
Aneurysm size	Small (<3.9 mm) Medium (4 – 10 mm) Large (>10 mm)	11 30 3
Median aneurysm size		5.01 mm

### 3.2. Image Preprocessing

**Figure 1:**
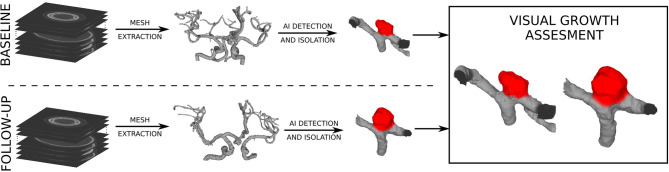
Preprocessing of angiographic scans to extract the surface meshes and the visual assessment of baseline and follow-up meshes to determine changes in (IA) morphology.

Preprocessing of the baseline and follow-up 3D images aimed to extract the surface mesh of intracranial vascular structures, i.e., vessels and aneurysms. First, vascular structures in the original CTA or MRA images were enhanced by applying the vesselness filter (Jerman et al., 2015). The original and filtered images were each interactively thresholded to mask vascular structures, and then the obtained masks were merged using *logical or* to get the segmentation mask. Possible spuriously masked voxel groups consisting of less than 60 connected voxels were removed by applying connected components filter to the segmentation mask. The resulting mask was fed into marching cubes and smooth non-shrinking algorithms (Lorensen and Cline, 1987; Cebral and Löhner, 2001) to extract the 3D surface mesh of intracranial vascular structures. The obtained meshes were visually assessed by the neurosurgeon to localize the IA(s). Next, manual mesh clipping tools were used to isolate each IA from its parent vasculature. The neurosurgeon visually inspected the corresponding pairs of isolated IA meshes from the baseline and follow-up scans, using side-by-side mesh visualization to determine morphology changes. The usual clinical procedure to determine aneurysm growth is to perform manual measurements separately on baseline and follow-up volumetric images, by observing and annotating 2D slices of 3D images. We used the side-by-side mesh visualization to give the neurosurgeon more insights into the change in morphology between baseline and follow-up image, which can not be interpreted only by observing 2D slices or using morphologic indices. Each baseline IA was thus classified as growing or stable. The sequence of applied preprocessing steps is shown in Figure 1.

### 3.3. Morphological Features

**Figure 2:**
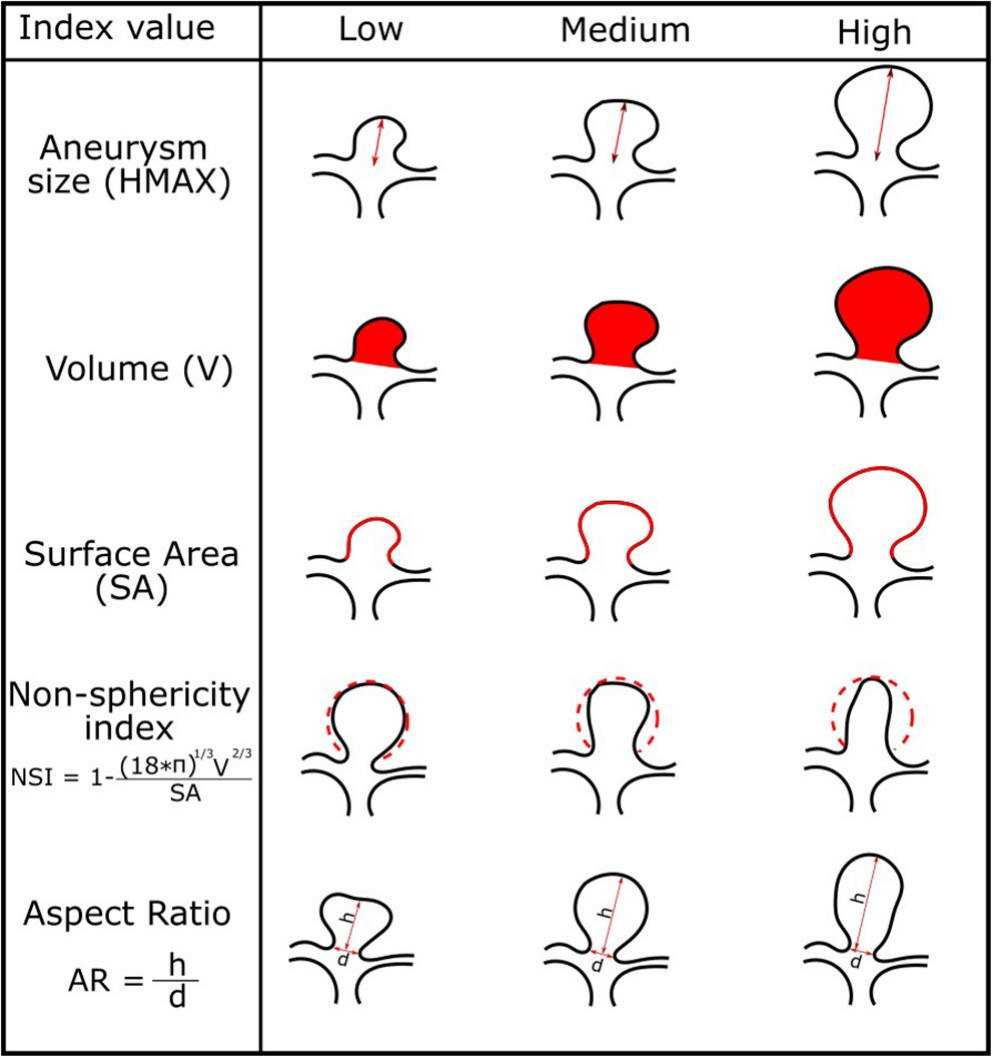
Morphologic indices of the IAs and corresponding illustration of IAs for low, medium, and high values of each index.

Quantification of IA morphology is often based on established *in-vivo* measurements such as HMAX, V, SA, NSI, and AR. These were determined as important factors for assessing rupture risk (Villablanca et al., 2013; Brown Jr and Broderick, 2014). For the purpose of future IA growth prediction, we computed the five features from the isolated IA surfaces. Figure 2 illustrates how the feature values are defined and computed.

### 3.4. Prediction Models

**Figure 3:**
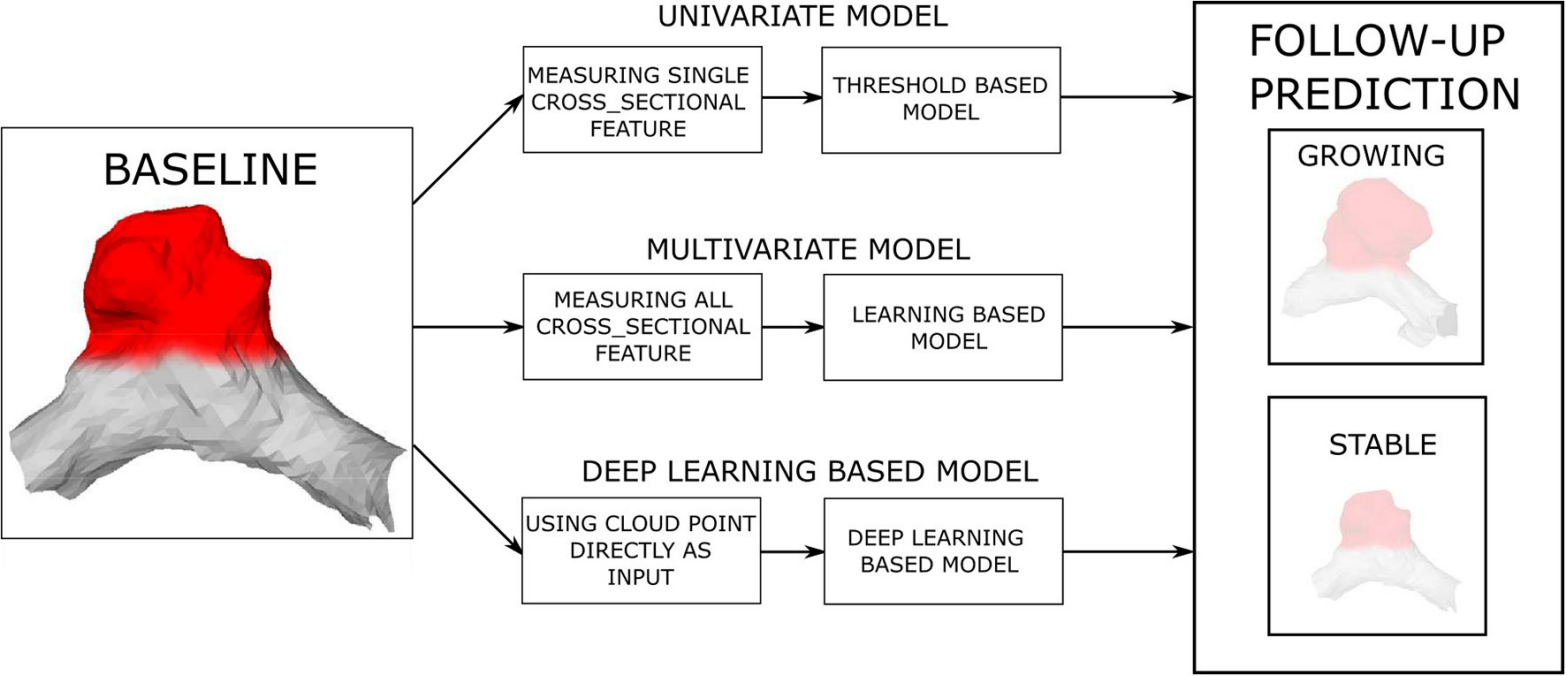
Prediction of future (AI) growth is based on the baseline IA morphology. Three distinct prediction approaches and several models were tested for the task.

This study hypothesizes that IA morphology (size, shape), as characterized by the baseline IA mesh, is a predictor of future AI growth. Therefore, we trained and validated prediction models of future artificial intelligence (AI) growth by using the baseline IA mesh and its morphological features as the input to the prediction models and the expert classification obtained by visual assessment of the follow-up scans as the model output. Three different types of prediction models were considered: (i) univariate threshold-based, (ii) multivariate learning, and (iii) mesh-based deep learning models. The models were distinguished by their increasing complexity and the input information (Figure 3). The univariate and multivariate models input the established (hand-crafted) features, such as HMAX, V, SA, NSI, and AR, computed from the baseline IA mesh, while the deep learning model input the extracted baseline surface mesh coordinates.

The univariate models applied a threshold to the input baseline IA morphological feature to distinguish between growing and stable aneurysms. This approach was based on the assumption that a particular feature takes on distinct values for the classes of growing and stable IAs. For each feature, the optimal threshold was determined by testing thresholds across the min–max range of a particular feature and choosing the value with maximal classification accuracy.

The morphological features may incorporate complementary information, which could benefit the classification task. Thus, we applied two multivariate learning models that aimed to find an optimal feature subset or feature weights and thus maximize classification performance. First was the random forests (RF) classifier (Breiman, 2001), which constructed a collection of binary decision trees from multiple random subsets of input features. In the training phase, optimal binary decisions in tree nodes, using feature thresholding similar to the univariate models, were found by maximizing the information gain. We used 100 trees, each with depth 3 to train the classifier. In the test phase, posterior class probabilities at leaf nodes were aggregated across the collection of binary decision trees to determine the most probable output class.

Another popular multivariate learning model is the multi-layer perceptron (MLP) (Rosenblatt, 1958), which is formulated as a hierarchical stack of ensemble linear classifiers, each with a non-linear activation. Learning the MLP was based on the backpropagation of the cross-entropy classification error on training samples, which iteratively updated the linear weights of the linear classifier such that classification error was reduced. The MLP classifier had two hidden layers, each with 100 neurons and was trained with a learning rate of 10^−4^ and LBFGS solver. We used all five features HMAX, V, SA, NSI, and AR as input to the RF and MLP models.

Recent deep learning approaches enable automatic feature extraction from raw input data. To apply this capability to the extracted surface meshes, we used the PointNet++ (Qi et al., 2017a,b). The PointNet++ inputs the raw point coordinates $p_{i}\;=\;{\left[x_{i},y_{i},z_{i}\right]}^{\text{T}}$ of the surface mesh and corresponding surface normals $n_{i}\;=\;{\left[n_{x,i},n_{y,i},n_{z,i}\right]}^{\text{T}};i\;=\;1,\ldots ,N$. Hence, the input surface mesh was represented as a 6 × *N* matrix. In the first step, the PointNet++ used a sequence of sampling and grouping layers at multiple resolutions, and a sequence of MLP layers to encode the pattern of the local regions into feature vectors. Specifically, a shared MLP used input rotation augmentation and then mapped each sampled and rotated six-dimensional input vector to 64 features, followed by a second shared MLP that mapped the 64 features to 1, 024 dimensions. Then, max pooling was used to create a global 1, 024-dimensional representation of the input mesh. The obtained global representation, which was scale and rotation invariant, was passed to a three-layer MLP with a two-class output for the purposes of our prediction task. Each surface mesh was resampled to 2, 048 points for training and inference with the PointNet++. During training, the negative log-likelihood loss was minimized using an SGD optimizer with a momentum of 0.9, an initial learning rate of 0.005, and a decay rate of 0.0001. The batch size was set to 32 and number of epochs was 200. The PointNet++ model was tested with two different inputs: (i) surface mesh of the IA dome and its parent vasculature and (ii) surface mesh of the IA dome only.

### 3.5. Experiments

The 44 aneurysm cases were used to test the performances of the prediction models. We tested five univariate models, i.e., for each of the features HMAX, SA, V, NSI, and AR. For evaluating the RF, MLP, and PointNet++ models, we adopted a four-fold cross-validation approach. Namely, the dataset was split into four-folds, each consisting of 11 IA cases. For each model, training and testing were executed in four runs, each run using 33 IA cases (3-folds) to train the model and the remaining 11 IA cases (1-fold) for testing. The ratio between stable and growing IAs was approximately the same across all four folds. All models used the same fold split and the same hyperparameters in all four runs. The classification scores obtained from the testing folds were aggregated and used for evaluation.

For classification evaluation purposes, we plotted the ROC curve and computed the area under ROC curve AUC. We reported the highest achieved classification accuracy and corresponding sensitivity and specificity.

### 3.6. Implementation

All preprocessing of volumetric images was implemented in Python programming language, using the SimpleITK library (Lowekamp et al., 2013). Mesh editing such as smoothing, remeshing, and clipping was performed in MeshLab (Cignoni et al., 2008). Morphological features were automatically computed from the extracted and isolated aneurysm dome meshes using our in-house Python scripts, based on the trimesh (tri, 2019) and numpy (Harris et al., 2020) libraries. We used the RF and MLP implementations in Python library scikit-learn (Pedregosa et al., 2011) and the PointNet++ implementation provided by its authors (Qi et al., 2017a).

Experiments were executed on a Linux workstation with an 8-core Intel I7 processor, 32 GB system RAM, and NVidia GPU with 11 GB RAM.

## 4. Results

**Figure 4:**
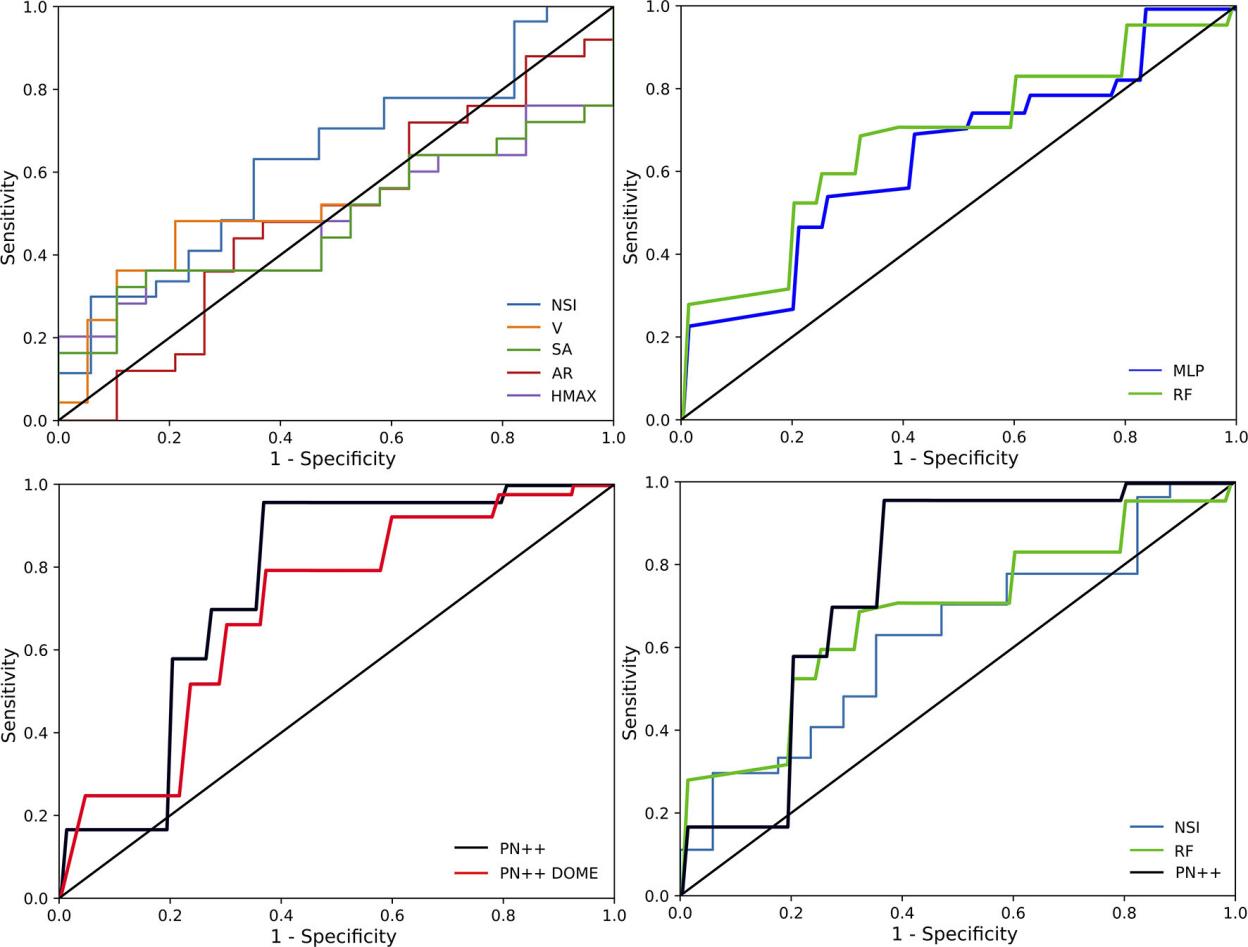
Receiver operating characteristics (ROC) curves for all tested prediction models separated in to four panels: ROC curves of univariate models (*upper left*), ROC curves of multivariate models (*upper right*), ROC curves of deep learning models (*lower left*), and best ROC curves for each approach (*lower right*).

Classification performances across all 44 IA cases and for the five univariate and three learning-based prediction models are summarized in Table 2. The ROC analysis for all methods is shown in Figure 4. In general, the univariate models exhibited poor performance; the classification accuracy was from 0.54 to 0.63 and the AUC values from 0.52 to 0.62. The best performing univariate model was based on the NSI (acccuracy = 0.63, AUC = 0.62), with a good sensitivity of 0.89, but a rather poor specificity of 0.29.

**Table 2:** Classification performance of future (IA) growth prediction models.

**Model**	**Test data**	**AUC**	**accuracy**	**sensitivity**	**specificity**
**NSI**	all	0.62	0.63	0.89	0.29
**HMAX**	all	0.52	0.54	0.36	0.80
**AR**	all	0.52	0.56	0.88	0.15
**V**	all	0.48	0.58	0.48	0.78
**SA**	all	0.48	0.56	0.36	**0.84**
**RF**	all	0.66	0.68	0.63	0.66
**MLP**	all	0.62	0.64	0.71	0.55
**PointNet++ (dome)**	all	0.72	0.77	0.80	0.63
**PointNet++**	all	**0.795**	**0.82**	**0.96**	0.63
**RF**	fold 1	0.56	0.55	0.33	0.80
**RF**	fold 2	0.81	0.81	0.83	0.8
**RF**	fold 3	0.63	0.64	0.66	0.6
**RF**	fold 4	0.63	0.72	1.0	0.4
**MLP**	fold 1	0.65	0.63	0.50	0.80
**MLP**	fold 2	0.61	0.63	0.83	0.4
**MLP**	fold 3	0.55	0.55	0.50	0.60
**MLP**	fold 4	0.67	0.72	1.00	0.4
**PointNet++**	fold 1	0.81	0.82	0.83	0.80
**PointNet++**	fold 2	0.70	0.73	1.0	0.4
**PointNet++**	fold 3	0.70	0.91	1.0	0.4
**PointNet++**	fold 4	0.75	0.82	1.0	0.5

*The best result across all data is marked in bold*.

Similar performances were observed with the multivariate learning-based MLP model, with an accuracy of 0.64 and an AUC of 0.62. Compared with the NSI based univariate model, the sensitivity–specificity (0.71 vs. 0.55, respectively) trade-off obtained by the MLP was more balanced. When using the RF, the trade-off was even more balanced with a sensitivity of 0.63 and a specificity of 0.66, and a slightly improved accuracy of 0.68 and an AUC of 0.66.

Deep learning-based PointNet++ model, using the IA dome and parent vasculature surface mesh as input, achieved the best classification performance with an accuracy of 0.82 and an AUC of 0.795. The respective values for the PointNet++ model input with IA dome surface mesh were slightly lower at 0.77 and 0.715. Hereafter, PointNet++ is used refer to the best performing model, unless otherwise stated. Compared to other tested models, the best PointNet++ model also improved on the sensitivity (0.96), while specificity was comparable with the RF model (0.63 vs. 0.66, respectively).

We also observed the performance of RF, MLP, and PointNet++ models in each of the four-folds (Table 2). In general, the model performances were quite comparable in terms of AUC, accuracy, and specificity, while the differences were mainly in the sensitivity, critical for detecting the future growing IAs. All methods had a sensitivity of 1.0 in fold number four, while the IA cases seem to be more challenging in the other three-folds. The PointNet++ model showed the most stable performance across all folds. This is also supported by the accuracy observed in the training folds, which was 0.97 for runs 1, 2, and 3 and 0.96 for run 4. The final loss was less than 0.07 in all four runs.

**Figure 5:**
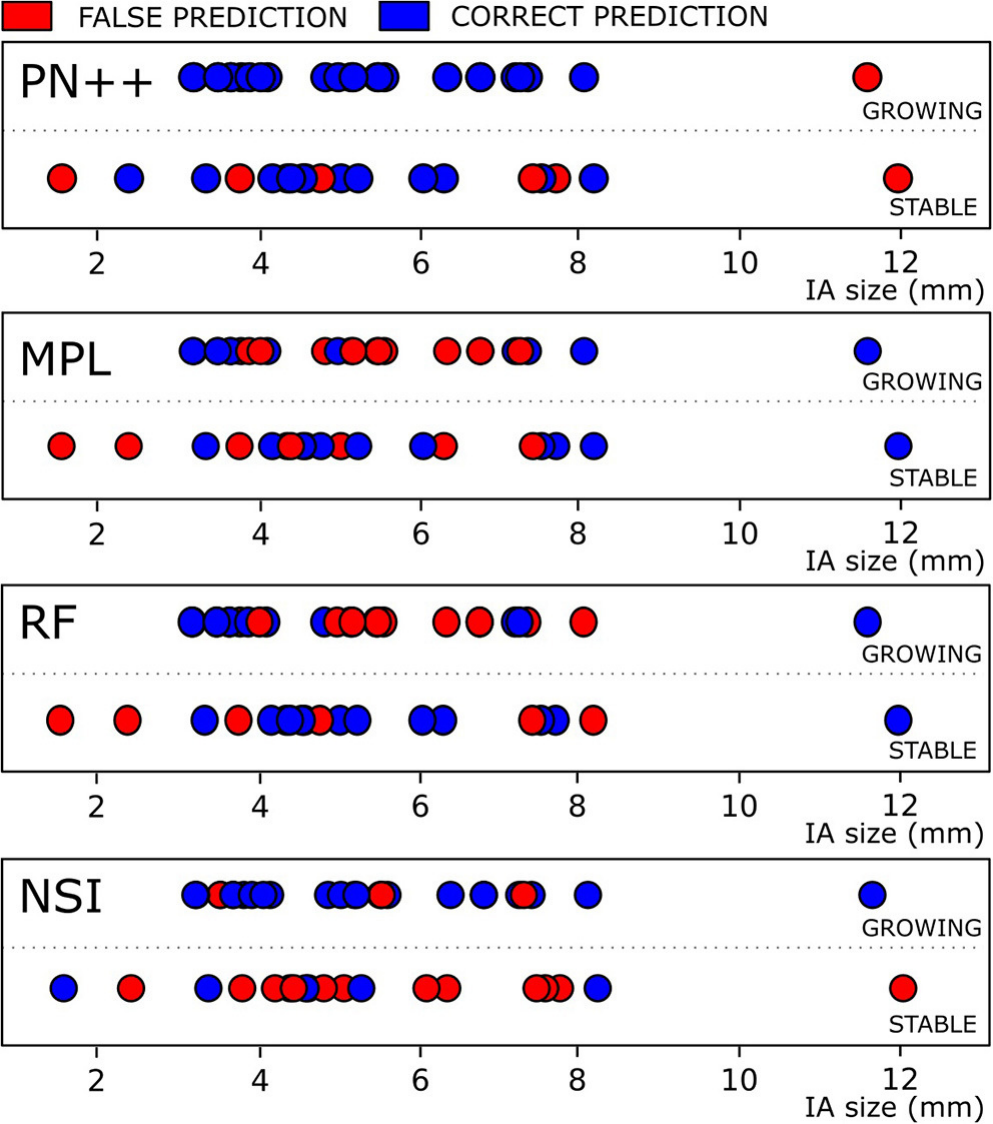
Classification results for IAs, classified as growing and stable based on follow-up image assessment, with respect to the baseline IA size are shown for four best-performing methods. *Blue and red dots* denote correct and false classification results.

Figure 5 visualizes the prediction results with respect to each ground truth class and the predicted class, and with respect to baseline IA size. In the stable IA class, 13 out of 19 IAs were correctly classified by PointNet++, while six falsely as growing IAs. More importantly, PointNet++ only misclassified one large IA (size > 10 mm) as stable, while it correctly classified 24 out of 25 growing IAs with sizes from 3 to 8 mm.

Other prediction models had a much larger proportion of misclassification in both stable and growing IA classes and throughout the whole range of the IA sizes.

## 5. Discussion

In this study, we objectively and comparatively evaluated the established and novel models for predicting future IA growth based on morphologic information. To our knowledge, this is the first study to consider deep learning models to predict future IA growth and employ only its baseline morphologic characteristics. In addition, we compared the deep learning-based approach with traditional learning-based methods and simple morphologic feature thresholding on our datasets. According to the results, the deep shape learning-based PointNet++ model can predict the future IA growth with high accuracy of 0.82, which is much higher than any of the established or non-deep-learning-based methods (accuracy ≤ 0.68).

The deep shape learning model successfully predicted aneurysm growth of 37 out of 44 IAs used in this study (Figure 5). The high sensitivity of 0.96 indicates that the model was capable of detecting those IAs that are prone to growth (24 out of 25 growing aneurysms were correctly classified). Specificity was mediocre at 0.63, namely, the model correctly identified 13 out of 19 stable aneurysms. In the terms of sensitivity, only the univariate thresholding based on the NSI feature came close (0.89). Interestingly, Chien et al. (2018) previously identified NSI as a relevant predictor of future AI growth. However, in this study, the NSI feature had poor specificity (0.29), which resulted in its overall mediocre accuracy of 0.63.

Univariate cross-sectional features other than NSI did not show a reasonable prediction value, which is also confirmed by the ROC curves in Figure 4. Namely, the models based on univariate features V, SA, AR, and HMAX have the ROC curves close to the diagonal, indicating a close to random classification performance. Both multivariate models, the RF and MLP, scored better accuracy (0.68 and 0.64, respectively) than the NSI-based model (0.63), or any other univariate based predictors. The slightly increased accuracy in the two models seems to be due to a better trade-off between sensitivity and specificity. In general, it seems that there is little complementary information in the five tested morphologic features, which the RF and MLP models could exploit. Another important aspect is that all five morphologic features characterize only the IA size and shape, but do not encode the information about the parent vasculature.

The proposed PointNet++ based deep learning model was superior compared to other tested methods, especially in classifying cases in the growing IA class. The proposed method was greatly improved on the state-of-the-art published results by Chien et al. (2018). In their study on 93 aneurysms, they achieved an AUC of 0.721, compared to the AUC of 0.795 by the PointNet++ model. There are two main benefits of using PointNet++. First, the PointNet++ inputs surface mesh vertex coordinates and corresponding normals and then aims to learn local shape features with an increasing contextual scale such that they are most discriminative for the posed classification task. For instance, the PointNet++ model trained on aneurysm dome surface mesh as inputs achieved better AUC than any univariate or multivariate models tested in this study, proving the importance of the automatically extracted local shape features. Second, another benefit of PointNet++ is that it may input both the aneurysm and its parent vasculature surface mesh, thus also taking into account local features of the proximal vessels and their configuration. There seems to be relevant additional morphologic information provided by the parent vasculature as the PointNet++ model trained solely on the IA dome surface mesh exhibited slightly inferior performances. Thus, an interesting avenue is to investigate the use of morphologic features that characterize the parent vasculature, such as aneurysm-to-vessel size ratio, aneurysm inclination angle, and vessel angle (Dhar et al., 2008). However, these features seem very crude and may not capture all the possible parent vasculature configurations. It does not seem reasonable to expect that such features could compete with the automated feature extraction mechanism in the PointNet++, which seamlessly weights the contributions of the IA dome and parent vasculature morphology for the posed classification task.

This study and similar studies (Liu et al., 2018, 2019) show that more accurate predictions may be obtained using machine learning models, compared to conventional univariate models based on a single morphologic feature. Liu et al. (2018) compared the morphology of ruptured and unruptured aneurysms and a trained machine learning-based model for predicting aneurysm growth. The authors achieved a high AUC of 0.948, which can also be attributed to the aneurysm morphology change due to rupture. Another study (Liu et al., 2019) based on the dataset of already ruptured aneurysms achieved an AUC of 0.853. Even though the datasets and the associated research question being addressed in those studies cannot be directly compared to ours, there is an obvious improvement when using machine learning-based methods compared to the use of conventional univariate morphologic features, possibly owing to the use of a rich feature set and larger training datasets. In this way, future studies could further improve the performances of models like RF and MLP.

An important limitation of this study is the number of IA cases in our dataset. Although follow-up imaging and “no treatment” approach is recommended for monitoring small IAs, it is not yet widely used in clinical practice. A few studies that used larger datasets usually had only extracted the manual measurements from aneurysm viusalizations, such as aneurysm size and other morphologic features (Chien et al., 2019), but unfortunately did not extract the surface meshes. Furthermore, since the clinical practice is in favor of surgical treatment, the availability of long-term follow-up imaging datasets is extremely rare. Despite these difficulties, we managed to retrospectively collect 44 pairs of IAs for this study and, by using a cross-validation approach, demonstrated a clear improvement in the prediction capability using vasculature surface meshes and the PointNet++ deep learning model for classification.

The high sensitivity of the proposed prediction model allows for adequate prioritization of surgical interventions for those patients at high risk of future aneurysm growth, thus mitigating the risk of spontaneous rupture in the waiting period. The observed specificity of the model is not very high; however, this does not seem to have adverse implications for clinical adoption. For example, in the current practice at our partnering clinical institution, most aneurysm patients will undergo surgical treatment and prioritizing patients according to certain risk measures is essentially the goal. Thus, the false-positive predictions, i.e., patients indicated as high risk but not exhibiting future aneurysm growth, would thus receive treatment earlier than otherwise. This is less concerning than the opposite scenario, where a false-negative result could lead to-high-risk patient not being treated. With the proposed PointNet++ model we observed a low number of false-negative results, leading to the high sensitivity, which is an important contribution of this study.

While the rate of growth and other surrogate measurements are highly relevant and can be assessed with high accuracy as shown in our previous study (Bizjak et al., 2019), the integration in the clinical routine proved to yield an insignificant impact, since, under current workflow and guidelines, the clinician rarely decides for the “no-treatment approach” with follow-up imaging. These practical observations motivated this study to provide prognostic tools to support the decision for the “no-treatment approach,” such that these tools are based solely on the baseline data and aim to determine future growth/rupture risk.

Causes of the rather low specificity of prediction models could lie in the distinctive aneurysm shape heterogeneity and homogeneity in the respective growing and stable classes and artifacts resulting from imperfect surface mesh extraction. For instance, future growing aneurysms generally exhibit higher values of NSI, indicating that the shape of such aneurysms is rather irregular. Shape irregularity is reflected through high variability in surface curvature and roughness, which are local surface features that the proposed PointNet++ model could capture extremely well. On the other hand, the class of stable aneurysms exhibits lower NSI values and seems much more homogeneous in terms of shape characteristics, with more regular convex shapes. However, the process of mesh extraction from the volumetric scans may introduce minor surface irregularities that could impact the prediction using the PointNet++ model, specifically by increasing the rate of false positives that adversely impacts the specificity. A possible strategy to improve specificity could thus be to increase the resolution of the volumetric scans and enhance their preprocessing, mesh extraction, and processing. Moreover, increasing the number of training cases might also further improve specificity and overall prediction performance as the PointNet++ model could better distinguish relevant local shape features from the artifacts when given more cases and thereby accordingly reweigh their contribution to the output classification score.

Correct classification of the growing IAs is most important for the clinical application of mitigating rupture risk and prioritizing surgical interventions. We thus feel that the proposed method can greatly change and improve the process of IA management in clinical practice, through providing rupture risk patient stratification with respect to IA growth prediction. Figure 4 shows that certain smaller IAs, predicted as growing, could be considered more urgently compared to larger, but predicted as stable, IAs. Furthermore, such prognostic tools may lead to wider clinical adoption of the imaging-based patient follow-up and render the “no treatment” approach as a viable alternative to surgical intervention in certain patients. Besides preventing ruptures during waiting periods, such an approach would indeed help to mitigate the high socio-economic impact arising due to the high incidence and prevalence of the IAs in the general population.

## Data Availability Statement

The datasets presented in this article are not readily available because; The datasets presented in this article are not readily available because the data set is the property of University Medical Centre Ljubljana, and we can not make it publicly available yet. Requests to access the datasets should be directed to Žiga Bizjak, ziga.bizjak@fe.uni-lj.si.

## Author Contributions

ŽB and ŽŠ designed this study. ŽB carried out the experiments and analyzed the data. ŽB, ŽŠ, and FP wrote and revised the manuscript. All authors contributed to the manuscript and approved the submitted version.

## Conflict of Interest

The authors declare that the research was conducted in the absence of any commercial or financial relationships that could be construed as a potential conflict of interest.
